# Within the Hospitals

**Published:** 1894-11-17

**Authors:** 


### The Institutional Workshop.

### WITHIN THE HOSPITALS.

By Our Special Commissioner.

### COTTAGE HOSPITALS: PRINCESS ALICE MEMORIAL HOSPITAL, EASTBOURNE.

Eastbourne is a delightful watering-place. The fine, invigorating quality of its air, the salubrity of its site, the efficiency of its local administration, the beauty of its roads, houses, and public gardens, and the magnificence of much of the scenery, all combine to make the stranger feel that there are few places where he can spend a holiday with greater benefit and more enjoyment. The railway journey to Eastbourne, owing to the improved service on the Brighton line, is now as easy as that to Brighton, and we are disposed to think, thanks in no small measure to the enterprise of the Duke of Devonshire, that no one who has given a fair trial to these two watering-places will have any difficulty in deciding in favour of Eastbourne. The hotels are excellently managed, replete with all necessary comforts, and the charges are, on the whole, reasonable. The arrangements made for the amusement of visitors throughout the year are varied and satisfactory; the opportunities for horse exercise and excursions are exceptionally good, and the sea-front offers attractions, which are certainly surpassed by few if any watering-places on the English coast. The view of the Downs from the hospital gate is one which lingers in the memory, and makes the denizen of a great city realise how much more fortunate is the lot of a sick person in a hospital like the Princess Alice Memorial Hospital than that of a patient in a great town institution, where the hospital site is necessarily surrounded, in the majority of instances, by houses and streets, the latter of which are alive with traffic during the greater portion of each twenty-four hours. No doubt there are counter-balancing advantages to the patients in the larger hospitals, which all must gratefully recognise, but when the stage of convalescence is reached, the additional comfort of residing in a beautiful building standing in its own grounds, from which magnificent views are obtainable, coupled with the purer air and greater brightness of the sunshine, must combine to invigorate the patient and hasten his recovery.

#### Interior of the Hospital.

Everything seems to have been done with a view to make the patients comfortable and secure their speedy recovery. The wards are well proportioned, airy, and bright. There is an adequate nursing staff, the members of which take a keen interest in the welfare of their patients, and the efficiency of everything within their jurisdiction. A generous confidence is placed in the patients, which tends to increase the efficiency and comfort of everybody in the hospital. The diet is liberal, the cooking excellent, and the quality of the food and the method of serving it up are better than those usually met with in hospitals of this size. The institution contains twenty-eight beds for adults and nine for children, or thirty-seven in all. There is an excellent children's ward, though, partly from want of funds but mainly from want of children to occupy them, the beds it contains are all vacant, and there seems no probability of their ever being utilised for the treatment of children. Such children as seek admission are treated in the general wards at the present time, and it seems to be desirable, seeing that a ward which is continuously unoccupied must necessarily get out of repair, that the committee should convert it into a general ward for the reception of adult patients.

#### Suggested Additions.

It is matter for regret and, we think, for criticism, that, although this hospital was built so recently as 1888, the drainage has proved so defective as to render it necessary to have it rearranged and renovated during the current year. At the present time there are no slop sinks in the hospital, and we venture to think that their introduction would lessen the work of the nurses and improve the hygienic condition of the wards. We also think that some of the governors who take an interest in the well-being of the institution might usefully present a portable dressing-tray of modern and approved pattern to each of the large wards. We would further suggest that some of the many ladies who are able to paint should undertake to decorate the ward lockers, as has been done at the Victoria Hospital in Guernsey. The pattern of the lockers is good, but the gentlemen might co-operate with the ladies in improving them by defraying the cost of adding marble tops to each locker, the absence of which is to be regretted. If some of the views in the neighbourhood were to be painted upon the lockers, the whole appearance of the wards would be greatly improved. We commend these suggestions to the ladies and gentlemen of Eastbourne, believing that they will meet with a ready and prompt response. We regret to find that none of the nursing staff have as yet joined the Royal National Pension Fund for Nurses, and we think the honorary secretary would render a good service to the hospital if he were to write to Dr. Perry, Guy's Hospital, and ask him to send a copy of the scheme which he has carried out at Guy's, by which that institution has become affiliated with the Pension Fund. The expense which would be entailed upon the committee might easily be met, and the bonuses thus given would be an encouragement to the nurses, and act as a stimulus to increased exertion on their part. Every well-administered hospital recognises the duty of encouraging the members of its staff to make provision against the day of sickness and incapacity. We are confident that many people who resort to Eastbourne with the object of improving their health would gladly subscribe to a fund, if such a fund be necessary, to enable the committee to pay a portion of the nurses' premiums on joining the Pension Fund. Mrs. Cameron, the matron, was away on a holiday at the time of our visit, but Miss Ramsay, an old Westminster nurse, who was acting in her absence, proved a most pleasant and efficient cicerone. Our visit was quite unexpected; it was made on a day and at an hour when visitors are not admitted, and we were pleased to find all the officers at their posts, and the whole institution working smoothly and efficiently.

